# Evaluation of MicroRNA92, MicroRNA638 in Acute Lymphoblastic Leukemia of Egyptian Children

**DOI:** 10.31557/APJCP.2021.22.5.1567

**Published:** 2021-05

**Authors:** Dina Fayed, Thoria Donia, Mohamed El-Shanshory, Ehab M M Ali, Tarek M Mohamed

**Affiliations:** 1 *Biochemistry Division, Department of Chemistry, Faculty of Science, Tanta University, Tanta, Egypt. *; 2 *Department of Pediatric, Hematology Unit, Faculty of Medicine, Tanta University, Tanta, Egypt. *; 3 *Department of Biochemistry, Faculty of Science, King Abdulaziz University, Jeddah, Saudi Arabia. *; 4 *Department of Chemistry, Faculty of Science, Tanta University, Tanta, Egypt. *

**Keywords:** Acute lymphoblastic leukemia, miRNA92a, miRNA638, RT-PCR

## Abstract

**Objective:**

miRNA considers a small non-coding RNA molecule that has tumor suppressor or oncogenic functions and regulates gene expression. miRNA may be involved in the pathogenesis of acute lymphoblastic leukemia (ALL). miRNA was evaluated in patients with ALL to correlate their importance in the clinical prediction and the response to chemotherapy.

**Subject and methods:**

The study population included 30 healthy control and 71 children with ALL is divided into 4 groups: healthy, newly diagnosed, remitted, and relapsed groups. We quantify miRNA 92a, miRNA 638 expression using real-time PCR in childhood ALL.

**Results:**

plasma miRNA 92a and miRNA 638 expressions were elevated in ALL cases at the time of diagnosis (2.51 and 2.19 folds), and relapsed (2.1 and 1.61 folds) than that of patients with remitted ALL. There was a positive correlation between miRNA 92a and miRNA 638 patients with ALL. Also, total leukocyte and blast correlated with miRNA 92a and miRNA 638 unlike hemoglobin, and platelets didn’t correlate with miRNA 92a and miRNA 638. The sensitivity of miRNA 92a and miRNA 638 were 41.5% and 54.7% respectively while the specificity was 100 % of miRNA 92a and miRNA 638.

**Conclusion:**

miRNA 92a and miRNA 638 are recommended to be used as potential predictive and follow-up markers in children with ALL remitted and relapsed cases.

## Introduction

The most common pediatric cancer is acute lymphoblastic leukemia (ALL), and it is developed due to the block of any lymphoid cell at a specific stage of development, containing primitive cells with potential for multilineage. Survival rates have improved in children with leukemia due to advances in treatment and supportive care (Al Azhar and Aisyi, 2021). At present, the diagnosis of ALL is based on immunophenotyping. Also, leukemia shows heterogeneity in treatment response and later risk of relapse. Therefore, new techniques are necessary to delineate the biomarkers of susceptibility and optimized therapy (Szczepanek, 2020).

miRNAs are noncoding small RNAs of 19 to 25 nucleotides with regulatory functions. miRNAs have crucial roles in some cellular processes, like differentiation, apoptosis, proliferation, and development, with inhibition of target gene(s) expression through inducing direct mRNA degradation, imperfect pairing with target mRNAs, or translational inhibition (Tian et al., 2017). miRNAs are derived from tumors and circulating in serum such as miR-21, miR-15b, miR-16, miR-24, miR92a, miR-155 and miR638 (Zavesky et al., 2016).

The miR-92a is a family of highly preserved miRNAs containing miR-25, miR-92a-1, miR-92a-2 and miR-363 that has identical seed regions. It arises from three distinct paralog clusters which are miR-17-92, miR-106b-25, and miR-106a-363 (Bai et al., 2019). Both miR-92a-1 and miR-92a-2 are processed to mature miRNA-92a. These miRNAs play a potent role in the regulation of blood vessel formation and mammalian organ development such as lungs, immune system, and heart (Li et al., 2014).

miR-638 has regulatory role in autophagy of esophageal squamous cell carcinoma (ESCC) and breast cancer cells. Overexpression of miR-638 promotes rapamycin- and starvation -induced autophagy (Ren et al., 2017). 

miR-638 acts as an oncogene in breast cancer cells and ESCC. Also, promotes proliferation and migration of cell as well as invasion in vitro and in vivo. miR-638 over expressed in ESCC and breast cancer tissues compared to normal tissues due to the regulatory mechanisms of miR-638 in autophagy (Ren et al., 2017).

In a previous study, the level of miR-92a level showed a significant decrease in both serum samples and tumor tissues derived from the patients with breast cancer than that of healthy control group due to the role it has in progression of cancer where associated with positive lymph node status and the ability for genes regulation in molecular pathways. miR-92a level was related to metastasis of lymph nodes and tumor size (Fang et al., 2017). Also, the miR-92a level was highly significant in both ALL and acute myeloid leukemia (AML) cells than in peripheral blood mononuclear cells from healthy subjects. In addition, miR-92a was expressed in ALL cells when compared to AML cells and patients with ALL miR-92a overexpression with poor prognosis (Ghai and Wang, 2016).

In a previous study of El-Halawani et al., (2014 ) found that plasma miRNA-92a was declined in AML patients when compared to healthy control.This downregulation is associated with poor response to induction chemotherapy regimen and poor risk cytogenetic group. 

Additionally, miR-638 is associated with early relapse in children with ALL, suggesting their possible association with prognosis (Xu et al., 2011) but it is never quantified in Egyptian children with ALL. This work aimed to evaluate miRNA 92a, miRNA 638 expression in Egyptian children with ALL as well as to correlate their importance in clinical prediction and therapeutic response.

## Materials and Methods

*Patients and methods*

*Ethics, consent, and permission*

The current study was carried out after approval of the research ethical committee, Tanta University (REC-Sci-TU-0016). 

*Registered individuals*

Totally 71 pediatric patients were presented at the Hematology / Oncology Unit, Pediatric Department, Tanta University Hospital, and Tanta Cancer Center with ages were ranged from 8 - 13 years. The subjects were divided into 4 groups: group I of 30 healthy children, group of II 32 children with newly diagnosed ALL, group III of 21 relapsed, and group IV of 18 remitted according to the pediatric protocol of therapy as following. 

The patients were injected Intravenous (IV) for the five weeks with 1.5mg/kg/m^2^/week Vincristine (VCR) and 25mg/m^2^/week Doxorubicin (DOX) (Lanzkowsky, 2011). Also, 10 doses asparaginase (ASNase) of 6,000 μ/ m was subcutaneously (SC) injected on alternate days, and 40mg/m^2^/day of oral prednisone for 6weeks. If no response is occurred with some patients, 100 mg/m^2^/dose IV etoposide was injected for three separate days as well as IV 750 mg/m^2^/dose cyclophosphamide (CP) with 100 /m^2^/dose IV of aracytin on the same three days and 5 g/m^2^ over 4 hours methotrexate (MTX) on day 28 (Pui et al., 2004; Lanzkowsky, 2005; El-Sharnouby et al., 2010 and Lopez-Lopez et al., 2013).

Next for 9 weeks firstly, IV MTX of 1gm/m^2^/dose every three weeks for 4 doses, and on specific days oral mercaptopurine of 60 mg/m^2^ daily, IV VCR of 1.5 mg/m^2^ IV with intramuscular (IM) pegylated ASNase of 2,500 units/m^2^ as well as IV CP of 750 mg/ m^2^/dose on two separate days (Chauvenet et al., 2007) with IV aracytin of 100/m^2^/dose and 36-39 and intrathecal (IT) MTX on certain days (Goldberg et al., 2003; Seibel et al., 2008).

IV VCR of 1.5 mg/m^2^ per day on five separate days with IV 100 mg/m^2^/dose MTX starting dose on day 0 and increased to 50 mg/m^2^/dose on days 10, 20, 30 and 40, IM pegylated ASNase of 2,500 units/m^2^ on days 1 and 21 and IT MTX on days 0 and 30.

10 mg/m^2^/day oral dexamethasone on two separate weeks with IV VCR of 1.5 mg/m^2^ 3 doses for two weeks, IM pegylated L-ASNase of 2500 u/m^2^ on day 4, IV DOX of 25 mg/m^2^ 3 doses for two weeks, IV CP of 1gm/m^2 ^on day 28, oral 6- thioguanine of 60 mg/m^2^ for two weeks, aracytin of 75mg/m^2^ on 8 certain days and one dose IT MTX and (Lanzkowsky, 2011).

For 30 months, IV MTX 20 mg/m^2^/ week in B cell ALL while in T cell ALL, the doses were 30 mg/m^2^, 120 mg/m^2^/day of prednisone for 5 days every 3 weeks, 2 mg/m^2 ^VCR IV every 3 weeks, with 50 mg/m^2^/day 6- mercaptopurine using oral route for two weeks and IT aracytin and MTX for 18 weeks (Goldberg et al., 2003).

This study has no children with liver diseases such as hepatitis B and hepatitis C virus and children autoimmune hepatitis was excluded from the study. Also, healthy children were registered and diagnosed with no signs of hematological disorders as confirmed by normal complete blood counts (CBC). Diagnosis of all children was carried out according to cytochemical and immunophenotyping criteria following Leukemia and Lymphoma World Health Organization classification.

*Sample collection*

Blood samples with 2 ml were collected from each subject on EDTA treated tubes then mixed well immediately and centrifuged for 15 minutes at 3000 rpm to separate plasma used for miRNA92a and miRNA638 evaluation in all study samples. Furthermore, complete blood picture using coulter counter was carried out on all both patient and control samples. 

*Molecular quantification of miRNA92a and miRNA 638 in plasma by RT-PCR*

miRNA extraction from plasma according to kit (mirVana PARIS kits - Ambion, Life Technologies, USA, #AM1556) protocol. cDNA synthesis with reagents was carried out according to the kit protocol (Quanti-Mir RT kit -SBI, System Biosciences, Cat. # RA420A-1). SYBR Green RT- PCR was used to assess the expression of plasma miRNA92a and miRNA638 with using miRNA16 as an internal control. The isolated cDNA was amplified using 2X Maxima SYBR Green/ROX qPCR Master Mix according to the manufacturer’s protocol (Thermo Scientific, USA, # K0221) using a specific forward primer for miRNA and a universal reverse primer included in the kit ( Quanti-Mir RT) (Table 1). 

*Statistical Analysis*

**Figure 1:**
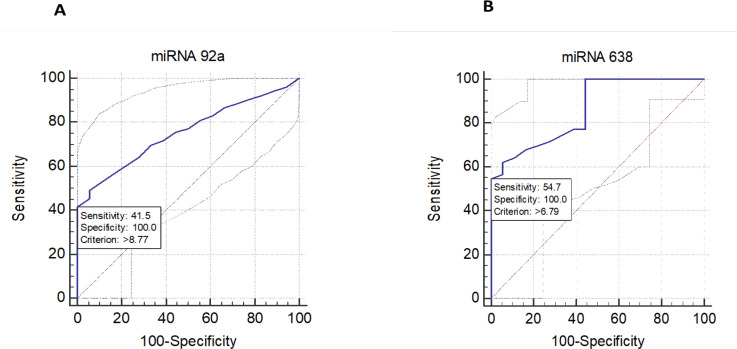
A: ROC curve for miRNA 92a (A) and miRNA 638 (B) in for ALL children sensitivity of miRNA 92a and miRNA 638 are 41.5 and 54.7 respectively

Group means ± SD were computed by student’s paired two-tailed t-test using GraphPad prism 6 software and P value of less than 0.05 was statistically significant. The correlation coefficient was calculated between different parameters by Excel. Receiver operating characteristic (ROC) curve was drawn by Minitab 18 (Figure 1) and used for calculation of area under the curve (AUC), sensitivity, and specificity of each investigated miRNA. 

## Results

As shown in Table 2 hemoglobin (Hb) level showed a highly significant decrease and the total leukocytic count (TLC) was highly significantly elevated in the newly diagnosed and relapsed as compared with the control or remitted group. Hb level showed a highly significant decrease and TLC was elevated in newly diagnosed as compared with the relapsed group. The platelets count in relapsed showed a high significance than that of the control group. Controls subjects had normal marrow with no blasts. Blasts percentage showed a significant elevation in newly diagnosed and relapsed groups than that of the remitted group. Also, the percentage of the blast showed a significant increase in newly diagnosed as compared with the relapsed group (Table 2). 

miRNA92a showed a 17.89, 7.13 and 14.72- folds increase, and also miRNA638 showed a 9.92, 4.54 and 7.32-folds increase in newly diagnosed, remitted, and relapsed groups respectively as compared with the control group. miRNA92a and miRNA638 showed a highly significant decrease in patients with relapsed (1.22 and 1.35 folds) and remitted (2.51 and 2.19 folds) when compared with newly diagnosed groups. Also, there was a significant increase in miRNA92a and miRNA638 in patients with relapsed by (2.1 and 1.61 folds) as compared with the remitted group. The ratio of miRNA92a to miRNA638 in the relapsed group was significantly increased when compared with the newly diagnosed group (Table 3).

*Diagnostic efficacy of miRNAs among childhood ALL groups*

AUC of plasma miRNA 92a (0.755) with 41.5% sensitivity and 100% specificity whereas plasma miRNA 638 showed an AUC of 0.862, sensitivity and specificity of 54.7% and 100 % respectively (Figure 1 and Table 4).

*Correlation between estimated miRNAs and clinical markers*

The correlation between the investigated miRNAs gene expression showed a positive significant correlation between miRNA 92a and miRNA 638 patients with ALL (Table 5).

miRNA92a reported a positive correlation with both TLC and blast (Table 5). While was not correlated with hemoglobin and platelets (Table 5). Hematological markers, both TLC and blast showed a significant correlation with miRNA638 (Table 5) on the contrary hemoglobin and platelets were not correlated with miRNA638 (Table 5).

**Table 1:** Primers Sequence for Real Time PCR

Gene	"Primer sequence (/5 ------ /3)"
*miRNA92a*	CTCAACTGGTGTCGTGGAGTCGGCAATTCAGTTGAGTCAGGCCG
*miRNA638*	GTCGTATCCAGTGCGTGTCGTGGAGTCGGCAATTGCACTGGATACGACAGGCCGC
*miRNA16*	CGGTAGCAGCACGTAAATATTGGCGA

**Table 2:** Hemoglobin level, TLC, Platelets Counts and Percentage of Blast Cells in Different Groups of Children with ALL

Groups/ Parameter	GI (Control) n=30	GII (Newly diagnosed) n=32	GIII (Remitted) n=18	GIV (Relapsed) n=21
Hb (mg/dl)	12.26 ± 0.51	5.25 ± 0.57^a, c^	11.96 ± 0.69^b, c^	8.2 ± 1.5 ^a, b^
TLC (×10^3^/mm^3^)	7.19±0.75	61.56 ± 46.15 ^a,c^	6.03±1.03 ^a,b,c^	18.22±17.16 ^a, b^
Platelets (×10^3^/mm^3^)	262.3±34.13	299.8±55.76^a, c^	299.3±61.42^a^	329.9±76.38^a^
Blast (%)	0	55.47±24.89^c^	1.61±0.77^b, c^	39±25.98^b^

Data are expressed as mean ± SD; Hb, Hemoglobin; TLC, total leucocyte count; ^a^, significant p value versus control; ^b^, significant p value versus newly diagnosed group; ^c^, significant p value versus relapsed group

**Table 3:** Fold Change for Expression of miRNA92A and miRNA 638 as Well as miRNA92a/ miRNA 638 Ratio of Children with ALL versus Control Children

Groups/ Parameter	GII (Newly diagnosed) n=32	GIII (Remitted) n=18	GIV (Relapsed) n=21
miRNA92a	17.89±7.9	7.13 ±0.93^a,b^	14.72±3.8^a^
miRNA638	10.19±4.11	4.54 ±1.69^a,b^	7.32±1.25^a^
Ratio miRNA92a/638	1.75 ±0.64	1.57 ±0.56^a,b^	2.01 ±0.77^a^

Data are expressed as mean fold change ± SD. ^a^, significant p value versus newly diagnosed group; ^b^, p value versus relapsed group.

**Table 4:** ROC Curve Analysis, Sensitivity, Specificity, Positive Predictive Value and Negative Predictive of miRNA Expression

Parameter	AUC	Sensitivity (%)	Specificity (%)	Positive predictive value (PPV) (%)	Negative predictive value (NPV)(%)	Cut off Value
miRNA92a	0.755	41.5	100	100	36.7	8.77
miRNA638	0.862	54.7	100	100	42.9	6.79

**Table 5:** Correlation between miRNA Expressions in Children with ALL and Their Clinical Analysis

Parameter	miRNA92a	miRNA638
miRNA92a	-----	r = 0.955
		p˂0.0001
miRNA638	r = 0.955	-----
	*p˂0.0001*	
Hb (mg/dl)	r = -0.789	r = -0.758
	*p˂0.0001*	*p˂0.0001*
TLC (×10^3^/mm^3^)	r = 0.547	r = 0.554
	*p˂0.0001*	*p˂0.0001*
Platelets (×10^3^/mm3)	r = -0.075	r = -0.076
	*p=0.225*	*p= 0.243*
Blast (%)	r = 0.588	r = 0.579
	*p˂0.0001*	*p˂0.0001*

Significant values (p<0.05); r, Correlation coefficient; TLC, total leukocytic count

## Discussion

Some of the miRNAs may act as tumor suppressor genes and the others act as tumor genes. Sometimes, some of the miRNAs can act as both, it depends on where they are being expressed in tissues (Misiewicz-Krzeminska et al., 2019). Because miRNA-92a is always expressed in many cells and it is an important molecule for the proliferation of both cells and cancer cells, its expression profiles will form a reliable part of diagnosis and detection of disease (El-Halawani et al., 2014), the present study investigated the expression of miRNA92a and miRNA 638 in plasma and its correlation with other clinical markers.

Our results showed that both plasma miRNA 92a and miRNA 638 expressions were elevated in ALL cases at the time of diagnosis (2.51 and 2.19 folds), and relapsed (2.1 and 1.61 folds) than that of patients with remitted ALL. At the same time, miRNA 92a and miRNA 638 expressions were lowering in the patients with relapsed ALL than their expression in newly diagnosed. These results were similar to another study by Tanaka et al. that revealed an elevation of miRNA 92a and miRNA 638 expressions in childhood with ALL in new diagnosis and relapsed group as compared with complete remission (Tanaka et al., 2009).This is because of the significant role of miRNA-92a as an oncogene and prognostic marker of ALL (Yu et al., 2019). 

Also, miRNA-92a was found to promote growth, metastasis, and chemoresistance in non-small-cell lung cancer cells by targeting Phosphatase and tensin homolog commonly known as PTEN (Ren ., 2016). Moreover, miR-92a overexpression induced the proliferation and invasion of cervical cancer cells by targeting F-Box and WD Repeat Domain Containing 7 (Zhou., 2015).This investigation suggesting that miR-92a plays a role as a carcinogenic miRNA in ALL.

The ratio of plasma miR-92a to miR-638 in all children samples in relapsed stage had significantly increased than that of children in complete remission. This is in accordance with another study of Ohyashiki et al who indicated that miRNA-92a significantly increased in acute leukemia patient’s plasma (Ohyashiki et al., 2010). Also, the ratio between plasma investigated microRNA was very useful to distinguish among leukemia patients in different stages (Tanaka et al., 2009).

Bhome et al., (2018) showed that many extracellular miRNAs are not bounded with exosomes/microvesicles but are instead binding to AGO2 protein in plasma that is generally thought to protect extracellular miRNAs and increase their stability in the extracellular fluids. This explains the elevation of miR92a and miR638 in plasma samples in our study. It seems that miR-92a has oncogenic potential. 

Also, our results showed a significant positive correlation between miRNA 92a and miRNA 638, suggesting a possible co-regulation between them in childhood ALL.

In addition, there was a significant negative correlation between miRNA92a and 638 expression and hemoglobin. This agreed with Szczepanek (2020) which revealed that leukemia impairs the ability of the bone marrow to produce red blood cells in childhood ALL. Also, there was a negative correlation between miRNA92a and 638 expressions and platelets but didn’t reach statistical significance (p= 0.225 and 0.243), this agreed with Ramzy et al., (2015).

miRNA92a and 638 expressions had a significant positive correlation with leukocytes in patients with ALL. miRNA92a and 638 expressions were decreased and correlated with decreasing TLC in remitted patients with ALL. These findings were in line with another study that reported the decreasing of TLC starts a few days after chemotherapy administration and reaches the lowest levels in the second or third week after chemotherapy. Moreover, there was a positive correlation between miRNA92a and 638 expressions and bone marrow blast percentage, this may be due to chemotherapy that kills any leukemia cells as possible and allows normal marrow cells to develop (Pui et al., 2014). 

Also, a recent study indicated that the miRNAs enhance leukemic cell proliferation, apoptosis inhibition, and an increase in cell resistance to chemotherapy drugs (Szczepanek, 2020). Our results suggest that miRNA92a have some oncogenic potential in pediatric ALL and might be used as a malignancy biomarker to predict the treatment response. Nowadays, there is great progress in chemotherapy drugs, but the resistance of tumor drug often leads to the failure of treatment. Relapse and drug resistance in leukemia cells have no reliable mechanism and may be due to deteriorated cell apoptosis by chemotherapy or a lack of drug efficacy (Mansoori et al., 2017).

In conclusion, the present study showed that plasma miRNA-92a may be used as a biomarker for the detection as well as in prognosis and relapse at diagnosis time of ALL in children. Also, due to its elevation in pediatric ALL, it may be used to predict the treatment response. However, more studies are needed to identify miRNA92a expression levels in different types and stages of childhood leukemia. It is evident that our findings should be validated further by multicenter trial using big sample size. 

## Author Contribution Statement

Contributing to this work was Dina Fayed, who collected samples after being diagnosed and supervised by Mohamed El-Shanshory. The Experimental parts were carried out by Dina Fayed and supervised by Tarek Mostafa Mohamed and Thoria Donia. The paper was written and revised by Ehab M. M. Ali, Mohamed El-Shanshory , and Tarek Mostafa Mohamed.
